# 646. Increasing Use of Interferon-Gamma Release Assay to Test for Pediatric Tuberculosis in a Low-Burden Setting

**DOI:** 10.1093/ofid/ofab466.843

**Published:** 2021-12-04

**Authors:** Jeffrey Campbell, Mingwei Sun, Wei He, Gabriella S Lamb, Gabriella S Lamb, Mary Tabatneck, Don Goldmann, Vishakha Sabharwal, Thomas Sandora, Jessica Haberer

**Affiliations:** 1 Boston Children's Hospital, Boston, MA; 2 Massachusetts General Hospital, Boston, MA; 3 Boston Children's Hospital, Harvard Medical School, Washington, DC; 4 MD, Boston, MA; 5 Harvard Medical School, Boston, MA

## Abstract

**Background:**

The American Academy of Pediatrics recommends tuberculin skin tests (TSTs) or interferon gamma release assays (IGRAs) to test for tuberculosis (TB) infection in children ≥2 years old, and prioritizes IGRA testing in Bacille Calmette-Guérin vaccine recipients due to cross-reactivity. TSTs require a return visit, which frequently results in loss to follow up. Growing evidence supports accuracy of IGRA testing in pediatric patients, including young children, leading to calls for preferential use of IGRA over TST. We sought to evaluate trends in IGRA use in children over time.

**Methods:**

We identified all TB infection tests conducted in children 5-17 years old at 2 academic medical systems in Boston from October 2015–January 2021. TSTs were identified using medication administration records, and IGRAs were identified using laboratory records. We computed the proportion of tests per month that were IGRA and TST. We used Pearson correlation to determine the association between month of testing and proportion of tests that were IGRAs.

**Results:**

21,471 TB infection tests were obtained from 16,778 patients during our timeframe. Median age of testing was 13.4 years (IQR 9.2 – 16.2 years). During the study period, there was a significant increase in the monthly proportion of TB infection tests that were IGRAs (Pearson correlation coefficient 0.92, P < 0.001). The total number of tests performed per month also increased, with seasonal increases in testing in late summer and early fall and a substantial decline in testing early in the COVID-19 pandemic.

Tuberculosis infection tests and proportion IGRA.

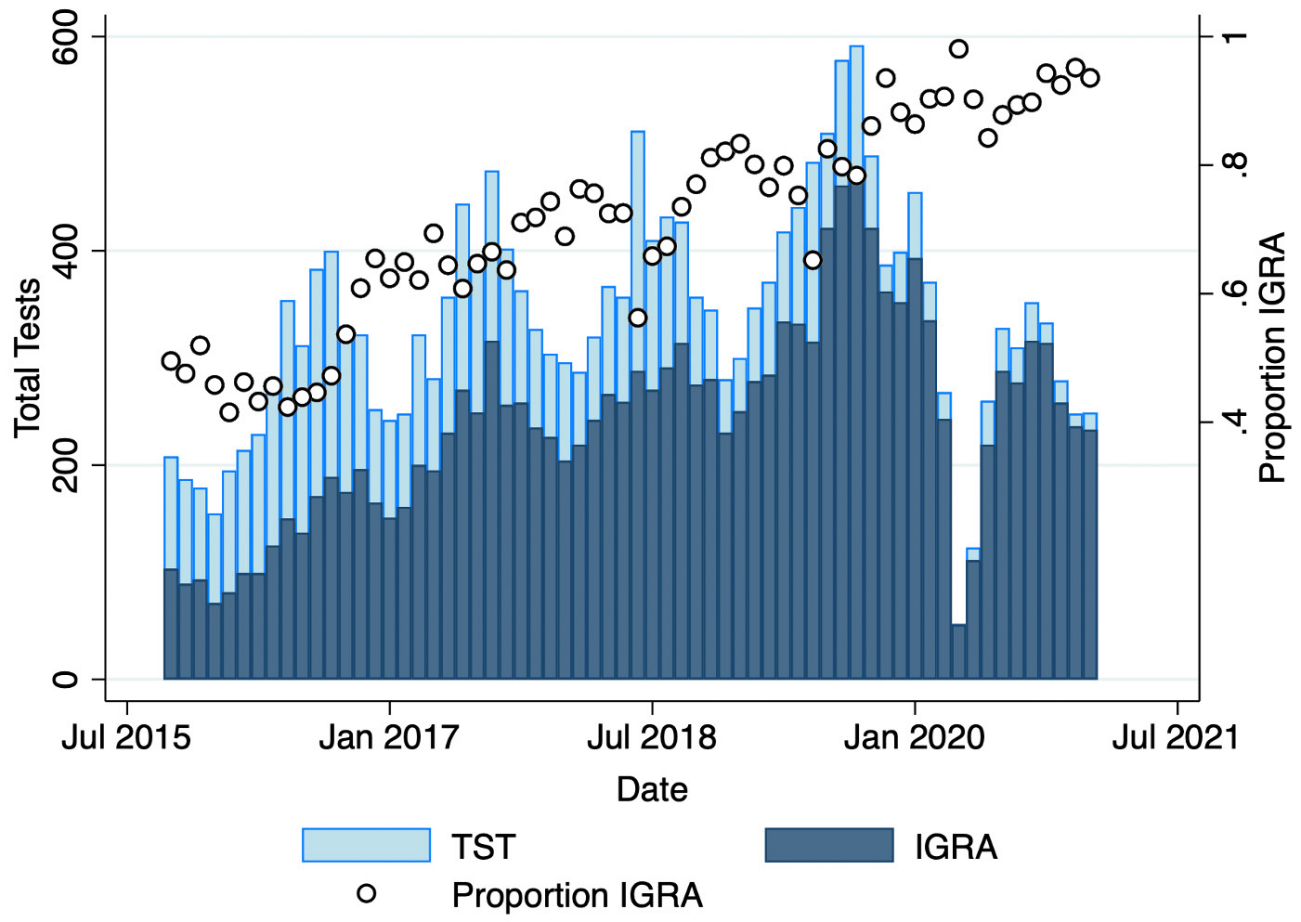

Total number of tuberculosis infection tests per month and proportion of tests that were interferon gamma release assays, from October 2015 - January 2021.

**Conclusion:**

Use of IGRAs among patients age 5-17 years of age increased significantly overall and compared to TST in two large Boston healthcare systems over a 5-year period. These results suggest a shift towards blood-based TB infection testing in a low-burden setting, which may improve completion of the pediatric TB infection care cascade. Future research is needed to determine reasons for changing testing modalities, and similar patterns in other settings.

**Disclosures:**

**Gabriella S. Lamb, MD, MPH**, Nothing to disclose

